# A Parallel Implementation of the Network Identification by Multiple Regression (NIR) Algorithm to Reverse-Engineer Regulatory Gene Networks

**DOI:** 10.1371/journal.pone.0010179

**Published:** 2010-04-21

**Authors:** Francesco Gregoretti, Vincenzo Belcastro, Diego di Bernardo, Gennaro Oliva

**Affiliations:** 1 Institute of High Performance Computing and Networking, Naples, Italy; 2 Telethon Institute of Genetics and Medicine, Naples, Italy; University of Maryland, United States of America

## Abstract

The reverse engineering of gene regulatory networks using gene expression profile data has become crucial to gain novel biological knowledge. Large amounts of data that need to be analyzed are currently being produced due to advances in microarray technologies. Using current reverse engineering algorithms to analyze large data sets can be very computational-intensive. These emerging computational requirements can be met using parallel computing techniques. It has been shown that the Network Identification by multiple Regression (NIR) algorithm performs better than the other ready-to-use reverse engineering software. However it cannot be used with large networks with thousands of nodes - as is the case in biological networks - due to the high time and space complexity. In this work we overcome this limitation by designing and developing a parallel version of the NIR algorithm. The new implementation of the algorithm reaches a very good accuracy even for large gene networks, improving our understanding of the gene regulatory networks that is crucial for a wide range of biomedical applications.

## Introduction

Microarray analysis methods produce large sets of gene expression data that can be exploited for novel insights into the fundamentals of molecular biology research. Inferring gene regulating networks from microarray gene expression data has become one of the major topics in system biology. Inferring or ‘reverse-engineering’ gene networks can be defined as the process of identifying regulatory gene interactions from experimental data through computational analysis.

Since the advent of microarray, various methods have been developed to infer the underlying gene regulatory network. The pioneer methods were simple clustering algorithms [1] where the similarity between genes were measured by a distance or “pseudo-distance” metric such as the clustering coefficient.

Since the structure to infer is a gene network, graphical models have been proposed and developed. This is the case of BANJO [2], that assumes that the gene network can be modeled as a Bayesian network. Bayesian statistics can be applied under some constrain [3].

Information theoretic approaches have been first proposed in [4] but the very first application was ARACNe [5]. ARACNe computes a pairwise pseudo-distance between each pair of genes to check their dependence. Theoretically ARACNe can be run to infer networks of any dimension.

All the mentioned algorithms have a ready-to-use software, and their performances have been tested and compared on both in-silico and in-vivo gene networks [6].

Here we will focus on an ODE-based algorithm, NIR [7], that relates the expression of each gene with the expression of other genes in the cell. It has been shown that NIR is able to correctly identify these relations called “influence interactions”. The ensemble of these interactions is referred to as gene network. Each of the recovered interactions within the gene network implies a regulatory interaction between components (Proteins, mRNAs, metabolites, etc.) of the cell. Gene networks can be used to identify the functional modules i.e. the subset of genes that regulate each other with multiple indirect interactions; to predict the response of the network to external perturbations and identify the genes directly hit; to identify physical interactions when integrated with additional information coming from sequence data and other experimental data.

Gene network inference algorithms based on ODEs relate gene transcript concentration changes to each other and to an external perturbation. The external perturbation is generally an experimental treatment that can alter the transcription rate of the genes in the cell. An example of perturbation is the treatment with a drug or a genetic perturbation that results in the overexpression or the downregulation of particular gene.

NIR algorithm achieves better results than the other network inference algorithms being able to reach peaks of 95% of correct predicted interactions. This has been shown in [8] where there have been selected and compared the algorithms capable of solving the network inference problem with an available ready-to-use software. In that work it wasn't possible to run NIR on data set with more than 100 genes because it would have taken too much time. Microarray technology allows the measurements of many thousands of transcripts at once and the mammalian gene regulatory network is of the order of 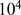. The complexity in handling networks of this size [6], motivated us to design, develop and run a parallel computing algorithm.

In section Methods we describe the NIR algorithm and its parallel implementation; in section Results we describe experimental results and in section Discussion we give our conclusions.

## Methods

### The NIR algorithm

It is possible to describe the gene network as a system of differential equations in which each equation describes the variation in time of the concentration of a particular transcript (gene), 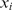, as a non linear function, 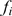, of the concentrations of the other transcripts:
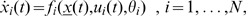
(1)where 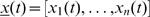 is a vector whose components are the concentrations of the transcripts measured at time 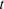, 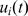 is the external perturbation applied at gene 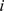 at time 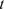, 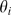 is a set of parameters describing interactions amongst genes, 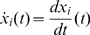 is the rate of change in transcription of transcript 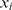 and 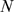 is the number of genes.

To reverse-engineer a network using ODEs means to choose a functional form for 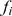 and then to estimate the unknown parameters 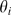 for each 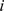 by using the gene expression data. With the ODE-based approach the resulting gene network will be a directed graph (i.e., if 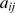 is the interaction between genes 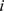 and 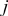, it specifies the direction of the interaction, that is, gene 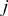 regulates gene 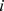 and not vice versa, 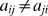).

We expand 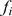 in a Taylor series around 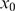, the point in which measurements were made. If we assume that perturbations around this point are sufficiently small, it is possible to truncate the Taylor series after the first order term, and obtain a linear expression:
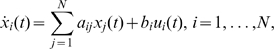
(2)where 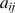 represents the influence of gene 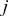 on gene 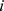, 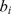 represents the effect of the external perturbation on 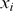 (in subsequent analysis, we set 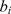 equal to one for the sake of simplicity) and 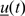 is the vector of the external perturbations at time 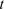 (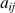 and 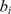 are the 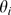 in equation (1)). 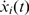 is the rate of change of concentration of gene 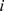 at time 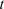, i.e., the first derivative of the mRNA concentration of gene 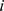 at time 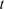. In proximity of the steady-state the concentrations of the 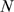 transcripts don't change in time (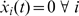) so that the (2) can be rewritten as:
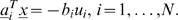
(3)Let us suppose that we have conducted 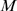 experiments such that we know the genes directly perturbed (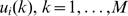) as well as the expression profiles following the experiments (transcript concentration levels from microarray data 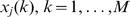). We can then solve the equation (3) for the unknown parameters 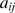, and thus obtain the ingoing edges per gene.

NIR applies the multiple linear regression method to estimate the unknown model parameters. It relies on the assumptions that the data 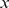 are realizations of a normally distributed random variable with known variances and the perturbations, 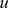, are random variables, also normally distributed with known variances. Generally, the response 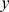 may be related to 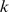 regressors and the model
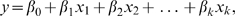
(4)is termed a multiple linear regression with 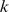 regressors.

Having 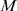 experiments (response observation points) at our disposal, the model for each gene of the network becomes:
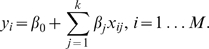
(5)The response (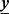) is given by the experimental perturbation values 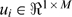, the regression variable values (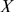) are given by the concentrations of the gene transcripts and the regression variable parameters are given by the components of the vector 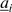, so that, in matrix form, the model becomes:
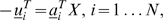
(6)with 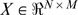. The regression analysis aims to best-fit the data by estimating the parameters of the model. NIR estimates the parameters of the regression variables for each gene, using the least squares method. These are the values for which the first derivative of the residual sum square function is zero:
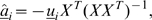
(7)under the assumption that the regressors are linearly independent.

Biological networks are sparse [9], thus NIR adopts the sparsity assumption that imposes an upper bound on the number of ingoing edges per gene (i.e. maximum number of regulators per gene), 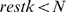, which can be chosen by the user.

For each gene the 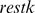 parameters that result in the smallest mean square deviation identify the 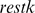 ingoing edges for that gene. The weight of the identified edges is given by the value of the estimated parameters. The choice of 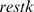 affects either the sensitivity to measurement errors or the execution time. A low value of 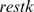 induces an increase in the solution sensitivity to measurement errors. A high value prohibitively increases the computational time needed to identify the regulatory network due to the high number of the regressor combinations to be included in the model. This number is equal to the number of combinations without repetitions of 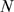 objects taken 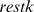 at a time:
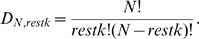
(8)This is polynomial of degree 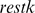 in the number of genes. The exhaustive approach which evaluates the regression for each combination is not feasible for gene networks larger than 100 genes (with 100 genes and 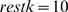 the number of combinations is of the order of 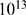), thus NIR uses the following heuristic approach.

For each gene 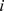:

- At the first step NIR computes (7) 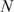 times by considering the regression variables one at time; the 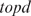 variables for which the sum of the squared deviations is minimized are selected as possible ingoing edges for the gene.
- At the second step NIR computes (7) by considering the remaining 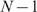 variables jointly with each of the first 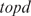 selected ones, that is 
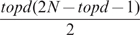
 (Gauss formula) pairs of variables; the 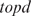 pairs of variables for which the sum of the squared deviations is minimized are selected as possible pairs of ingoing edges for the gene.
- At step 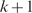 NIR computes (7) by considering the remaining 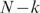 variables jointly with each of the 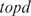 sets of 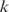 variables selected at the previous step, that is 
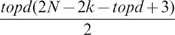
 sets of 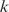 variables are considered; the 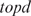 sets of 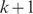 variables for which the sum of the squared deviations is minimized are selected as possible sets of 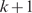 ingoing edges for the gene.
- The process ends when the number of regression variables selected reaches 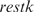; the set of 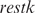 variables for which the sum of the squared deviations is minimized identifies the set of parameters 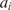 corresponding to the input regulations affecting expression profile of gene 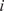.

The final output is an adjacency matrix, where each element is the edge 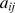, that encodes the directed graph. The number of times (7) is calculated for each gene is 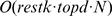, so the overall number of times (7) is calculated is 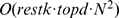. The computational complexity of (7) at step 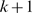, for the submatrix of 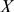 whose rows correspond to a set of 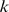 variables (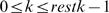), is 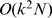. The overall computational complexity is therefore 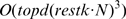.

### The NIR parallel version and its implementation

The NIR algorithm can be easily parallelized to handle large problems in a computationally efficient manner by distributing the overall computational burden among different processors to reduce the total execution time. In order to address the high computational cost issue of the NIR algorithm we have applied some specific implementation optimizations along with parallel programming techniques.

The computational core of the NIR algorithm is the equation (7) where X is a submatrix of the gene expression matrix composed only of 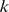 rows (with 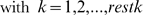). From the matrix-matrix product definition applied to the submatrix 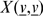, with 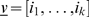 vector of 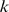 indices, it follows that:
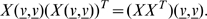
(9)Therefore for each step of the algorithm, we don't compute any matrix-matrix product operation 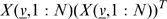. On the contrary the product 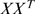 is computed once and for all at the beginning of the program. At each step 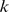, our implementation just selects the symmetric submatrix of 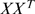 whose row and column indices correspond to the 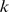 possible ingoing edges for the gene. Let 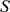 be this submatrix of dimension 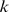 stored in packed format.

In each experiment only one gene is perturbed. This implies that for each gene 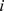 the perturbation vector 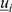 is equal to 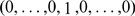 and then the product 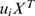 reduces to the 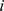-th row of 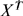. Denote this row by 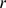.


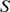
 is positive definite so we can apply the Cholesky factorization to the matrix 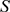 in order to compute 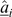 as solution of the system of equations 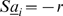, thus avoiding the matrix inversion. We rely on the LAPACK [10] routine DPPSV to solve this system of equations with a computational complexity of 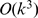.

By avoiding the matrix product in (7), the parallel algorithm complexity is decreased by one order of magnitude: at the generic step the computational complexity is 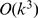, the overall computational complexity is therefore 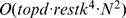


The parallelization is implemented by assigning different genes to different computing processes: each process takes care of 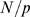 genes where 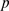 is the number of processes available. The computing steps described in the previous section can be performed independently for each gene so each process can compute the results for its genes independently without communication. The parallel algorithm has been implemented in C using the MPI standard.

## Results

We carried out two kind of tests: to measure the result accuracy of the algorithm and to measure the efficiency in terms of speed-up. In order to measure the result accuracy we ran the program, by using the ‘in silico’ data generated by [8], on 20 different networks counting 1000 genes, with 10 as average in degree per gene. ‘In silico’ data are gene expression data generated by a computer model of the gene regulation that enable one to check the performance of algorithms against a perfectly known truth. For each network we generated 1000 experiments perturbing a different single gene at time (local steady-state data).

The program has been executed on 100 processors of an HP XC6000 Cluster with Itanium 2 biprocessors nodes and a Quadrics ELAN 4 network. On average it took 984 seconds to generate the results for each gene network. The program recovered most of the true interactions as shown in Table 1. We compared the results from the simulations obtained by our implementation with the ones obtained by the network inference software reviewed in [8], setting the parameters 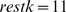, 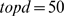. Some software infers the network just as an undirected graph (i.e. the direction of the interaction is not specified, 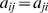), while NIR generates directed and signed graphs. In order to be able to compare the different types of software, we computed PPV and Se by first transforming the real (signed directed graphs) and inferred networks yielded by our implementation into undirected graphs (labeled 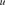 in Table 1). As shown in Table 1, NIR performs better than the other ready-to-use software even for 1000 gene networks.

**Table 1:** Results of the application of network inference algorithms on the simulated dataset.

Data Sets	ARACNe		BANJO		NIR		Clustering		Random
	PPV	Se	PPV	Se	PPV	Se	PPV	Se	PPV
Local (steady-state)									
10×10	0.53 	0.61 	0.41 	0.50 	0.63 	0.96 	0.39 	0.38 	0.36 
			0.25 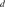	0.18 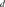	0.57 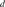	0.93 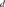			0.20 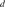
			0.15 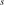	0.05 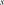	0.57 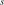	0.93 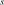			0.10 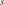
100×100	0.56 	0.28 	0.71 	0.00 	0.97 	0.87 	0.29 	0.18 	0.19 
			0.42 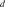	0.00 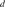	0.96 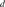	0.86 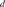			0.10 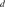
			0.60 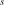	0.00 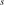	0.96 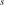	0.86 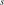			0.05 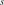
1000×1000	0.66 	0.65 	-	-	**0.91** 	**0.82** 	**0.20** 	**0.10** 	**0.02** 
					**0.84** 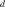	**0.84** 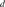			

PPV: Positive Predicted Value (or accuracy) defined as 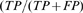, where 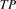 is true positive and 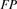 is false positive; Se: Sensitivity defined as 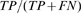 with 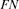 false negative. 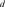: directed graph; : undirected graph. In bold are the results obtained by using our parallel implementation of the NIR algorithm which could not be obtained in [8]. NIR performs significantly better than other software even for the 1000 gene networks.

Moreover we ran the program on a 2500 gene network, even though we didn't have any other software predictions to compare the results with. We set the same values for the parameters 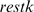 and 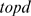 as before. It took around 12450 seconds to generates the results and we obtained the following values for PPV and Sensitivity: 0.26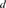, 0.27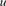 and 0.10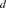, 0.11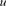 respectively. In order to measure the parallel efficiency we run the program for a 1000 gene network on different number of processors. The execution times and speedups are shown in Table 2. To evaluate the parallel speed-up, we developed a serial version of the algorithm with the implementation optimizations discussed in the previous paragraph.

**Table 2:** Total execution times in seconds.

number of procs	time (secs)	speedup
1	98896	-
10	9725	10.1
20	4798	20.6
40	2406	41.1
60	1643	60.2
80	1259	78.6
100	969	102.0

## Discussion

We have designed, developed and tested a parallel version of the NIR algorithm whose purpose is to infer gene regulating networks from microarray gene expression data. Our parallel algorithm reduces the time complexity of the original NIR algorithm by one order of magnitude by avoiding the useless repetition of matrix multiplication. The algorithm uses data parallelism, distributing the gene expression data over the available processors. The tests were performed on large networks (N = 2500) that couldn't be efficiently analyzed by the original serial version. The results confirm the improvements in accuracy, in terms of positive predicted values, and sensitivity, in terms of false negatives, with the respect to the other inferring methods. In addition, the parallel algorithm scales well as the number of processors increases and has a linear speedup.
